# A Transgenic Mouse Line Expressing the Red Fluorescent Protein tdTomato in GABAergic Neurons

**DOI:** 10.1371/journal.pone.0129934

**Published:** 2015-06-15

**Authors:** Stefanie Besser, Marit Sicker, Grit Marx, Ulrike Winkler, Volker Eulenburg, Swen Hülsmann, Johannes Hirrlinger

**Affiliations:** 1 Carl-Ludwig-Institute for Physiology, University of Leipzig, Leipzig, Germany; 2 Institute for Biochemistry and Molecular Medicine, University of Erlangen, Erlangen, Germany; 3 Laboratory for Experimental Neuroanesthesiology, Clinic for Anesthesiology, University Hospital Göttingen, Göttingen, Germany; 4 Center for Nanoscale Microscopy and Molecular Physiology of the Brain (CNMPB), Göttingen, Germany; 5 Department of Neurogenetics, Max Planck Institute of Experimental Medicine, Göttingen, Germany; CNRS—Université Aix Marseille, FRANCE

## Abstract

GABAergic inhibitory neurons are a large population of neurons in the central nervous system (CNS) of mammals and crucially contribute to the function of the circuitry of the brain. To identify specific cell types and investigate their functions labelling of cell populations by transgenic expression of fluorescent proteins is a powerful approach. While a number of mouse lines expressing the green fluorescent protein (GFP) in different subpopulations of GABAergic cells are available, GFP expressing mouse lines are not suitable for either crossbreeding to other mouse lines expressing GFP in other cell types or for Ca^2+^-imaging using the superior green Ca^2+^-indicator dyes. Therefore, we have generated a novel transgenic mouse line expressing the red fluorescent protein tdTomato in GABAergic neurons using a bacterial artificial chromosome based strategy and inserting the tdTomato open reading frame at the start codon within exon 1 of the GAD2 gene encoding glutamic acid decarboxylase 65 (GAD65). TdTomato expression was observed in all expected brain regions; however, the fluorescence intensity was highest in the olfactory bulb and the striatum. Robust expression was also observed in cortical and hippocampal neurons, Purkinje cells in the cerebellum, amacrine cells in the retina as well as in cells migrating along the rostral migratory stream. In cortex, hippocampus, olfactory bulb and brainstem, 80% to 90% of neurons expressing endogenous GAD65 also expressed the fluorescent protein. Moreover, almost all tdTomato-expressing cells coexpressed GAD65, indicating that indeed only GABAergic neurons are labelled by tdTomato expression. This mouse line with its unique spectral properties for labelling GABAergic neurons will therefore be a valuable new tool for research addressing this fascinating cell type.

## Introduction

Inhibitory interneurons are crucial components of the circuitry within the central nervous system (CNS) and disturbance of the balance between excitation and inhibition contributes to many diseases including epilepsy, schizophrenia and persistant pain [1–6]. The two main inhibitory neurotransmitters in the vertebrate CNS are γ-aminobutyric acid (GABA) and glycine, which are released by GABAergic or glycinergic cells, respectively. Furthermore, accumulating evidence suggests that some neurons use both transmitters simultaneously [7,8]. GABA is synthetized in GABAergic neurons by the decarboxylation of glutamate catalyzed by the enzyme glutamic acid decarboxylase (GAD). Two isoforms of this enzyme are known, which are referred to as GAD65 and GAD67. These isoforms are encoded by two distinct genes (GAD2 and GAD1, respectively) and the existence of two GAD isoforms is highly conserved among vertebrates, sharing more than 95% identity among the amino acid sequence of the cat, rat, mouse, and human [9–12]. Genetic deletion of GAD67 in mice leads to rapid postnatal death [13], while GAD65-/- mice develop spontaneous seizures and show an increased mortality [14,15]. GAD65 and GAD67 are both present in most classes of GABAergic neurons [16] but they are differently localized within the neurons. While GAD67 protein can be detected at higher levels in many GAD-containing cell bodies, GAD65 protein is mainly located in axon terminals [11,17–21] where it is functionally coupled to transport of GABA into synaptic vesicles [22]. In addition, the two isoforms show different interaction with the cofactor pyridoxal 5’-phosphate. Almost all GAD67 forms an active holoenzyme, saturated with its cofactor whereas only about half of GAD65 exists as active holoenzyme. It is suggested that GAD67 is capable of immediate GABA synthesis to allow prolonged high rates of firing, but might be also associated with a metabolic GABA pool. The GAD65 apoenzyme (enzyme without bound cofactor) might be a supply of inactive enzyme that can become active in response to increased demands on the GABAergic neuron [17–19,23,24]. In summary, GAD65 and GAD67 have been suggested to contribute to different functions within the neuron, but both isoforms are reliable indicators for the identification of GABAergic neurons and most GABAergic cells express both isoforms [16].

The visualization of specific cell populations in the brain by transgenic expression of fluorescent proteins driven by a cell-type specific promoter has greatly facilitated the analysis of the morphological and functional properties of these cells. In respect to inhibitory interneurons, mice have been generated expressing the green fluorescent protein EGFP e.g. in glycinergic cells driven by the GlyT2-promoter [25], in parvalbumin-positive neurons [26], as well as in GABAergic neurons using the GAD65 promoter [27] and GAD67 promoter [28,29]. In addition, mice have been generated with fluorescently labelled astrocytes [30,31], microglial cells [32], oligodendrocytes [31,33] as well as projection neurons [31,34]. Unfortunately, most of these transgenic mouse lines (including all lines labelling GABAergic neurons) express the green fluorescent protein, prohibiting the simultaneous labelling of two cell populations by different fluorescent proteins. Furthermore, as the most useful small molecular Ca^2+^-indicator dyes show green fluorescence (like e.g. Fluo4, Oregon Green BAPTA) it is difficult to combine these dyes with mouse lines expressing green fluorescent proteins.

To overcome these limitations, we generated a novel transgenic mouse line expressing the red fluorescent protein tdTomato in GABAergic neurons under the control of the GAD65-promoter. In this mouse line, almost all cells expressing tdTomato simultaneously express the endogenous GAD65 protein, indicating that indeed only GABAergic cells are fluorescently labelled. This mouse line will therefore be a valuable tool for labelling GABAergic neurons simultaneously to other cell populations both for *in situ* and *in vivo* experiments.

## Materials and Methods

### Ethics statement

Animals were bred in the animal facility of the Medical Faculty of the University of Leipzig and treated in accordance with the German Protection of Animals Act (TSchG §4 Abs. 3) and with the guidelines for the welfare of experimental animals issued by the European Communities Council Directive of 24. November 1986 (86/609/EEC). Experiments were approved (registration number TVV66/12) by the Tierschutzkommission (Institutional Animal Care and Use Committee) of the Faculty of Medicine, University of Leipzig, as well as by the local authorities (Landesdirection Leipzig). Mice were housed in a 12h/12h light dark cycle with access to food and water ad libitum and deeply anesthetized with diethylether before sacrificing the animals.

### Cloning of the transgene construct

**Fig 1:**
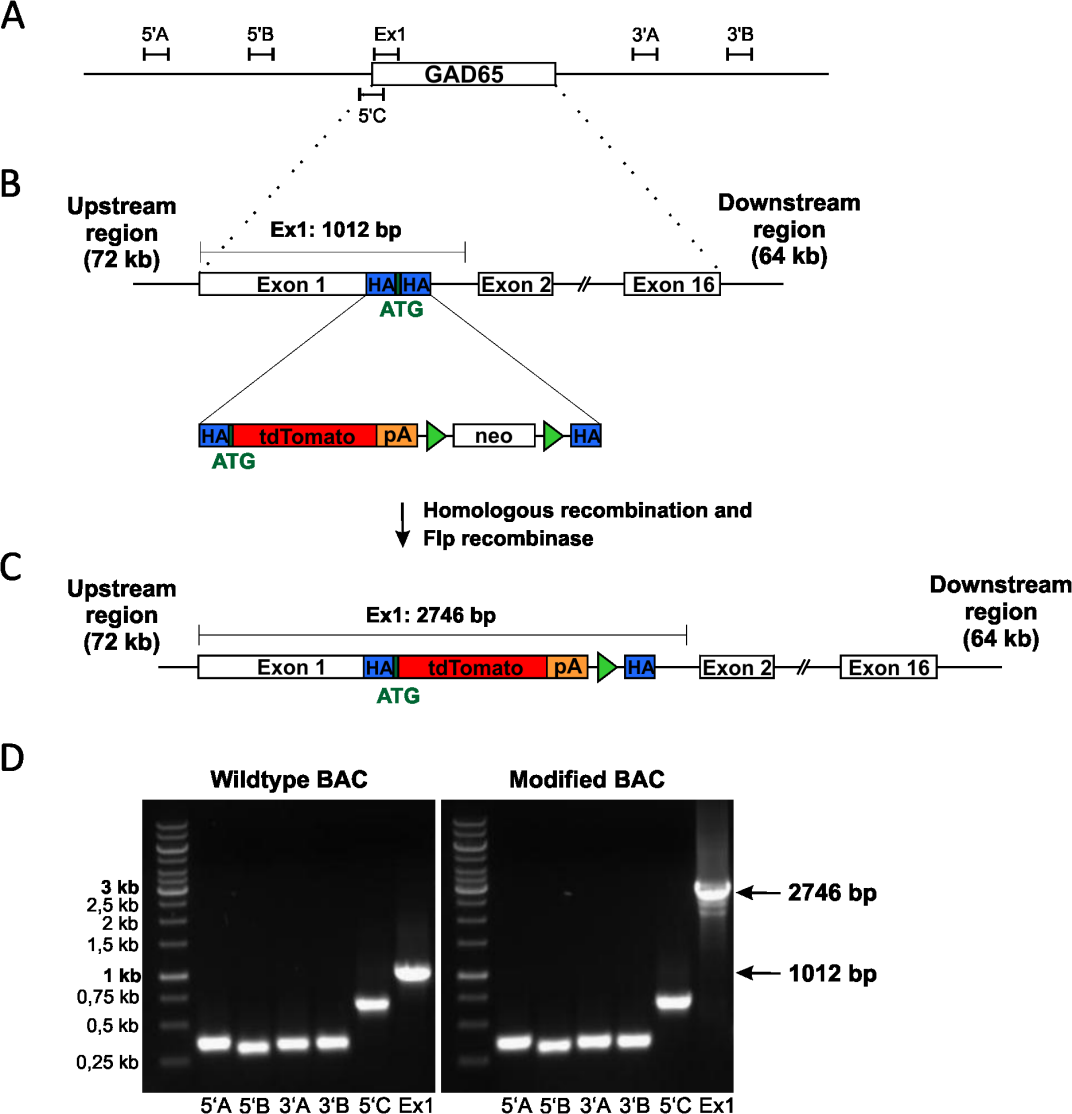
Strategy for the generation of GAD65-tdTomato mice expressing the fluorescent protein tdTomato under control of the GAD65 promoter in GABAergic neurons. **A:** Schematic representation of the BAC clone RPCI 23-407K8 (208,325 bp in total) containing the mouse full-length gene GAD65. Locations of PCR products for BAC verification (5’A-C; 3’A-B; Ex1) are indicated. **B:** Structure of the wild type GAD65 gene as well as the targeting construct. The endogenous start codon is located within exon 1. PCR reaction with primers (Ex1) flanking exon 1 results in a DNA fragment of 1012 bp in the wild type gene. The transgene consists of the ORF of tdTomato, a SV40-PolyA site as well as a FRT-flanked neomycin resistance cassette (neo) and was inserted directly after the endogenous ATG using homologous recombination provoked by homology arms (HA) indicated. **C:** Representation of the modified BAC containing the GAD65-tdTomato transgene after removal of the neomycin resistance cassette using Flp recombination. PCR using the same primers (Ex1) flanking exon 1 results in a product of 2746 bp in the modified BAC. **D:** PCR verification of the identity and integrity of the BAC using primers located 5‘ and 3‘ of the GAD65 gene (5’A-C; 3’A-B) as well as verification of the targeted modification site using primers spanning exon1 (Ex1).

The BAC clone 407K8 from the mouse C57/Bl6 BAC library RPCI-23 (Research Genetics Inc., Huntsville, AL) containing the GAD2 (GAD65) gene was identified by using the ENSEMBL database of the Wellcome Trust Sanger Institute (http://www.ensembl.org). It comprises 208,325 bp (base 22,549,829 to 22,758,153 of chromosome 2) including all known 16 exons of the GAD65 gene, 72 kb of the 5’ upstream region containing the presumptive GAD65 promoter elements as well as 64 kb 3’ downstream of exon 16. The BAC clone RPCI 23-407K8 was obtained from Source Bioscience imaGenes (Berlin, Germany). The presence of the GAD65 exon 1 coding sequence within BAC 407K8 as well as the integrity of the BAC clone was verified by polymerase chain reaction (PCR) and sequence analysis of exon 1 and selected areas in the 5’- and 3’-regions of the BAC (Fig 1A; Ex1 s: CGCCTGTGGCTGGGTCAGCA, Ex1 as: GCAGCTCACAACGCCGCCCTT, 5’A s: TGTATATCCTTGGACCAGGGAGTGGC, 5’A as: GCAGTACCCCACTCCCAGTACCAA; 5’B s: TCTTCCTCCCAAAGTCCTAGACCTCCA, 5’B as: TGGGTTTCCGAGGTCGTCTTGGAG; 5’C s: AGAACCACTTTTGCTCGGCAGCAG, 5’C as: CATGGGTTCTGCTAGACTGGCG; 3’A s: CACCCATCCCTGGACAAGTGGC, 3’A as: GCACCGTCTGTTTGGTCTGTGG; 3’B s: CATCTGCCTGCCCCTCAACTCC, 3’B as: TCCGCTGCACTCACTCTTGTCAGT).

The tdTomato open reading frame followed by SV40 polyadenylation sequence derived from the expression vector pCMV-tdTomato (Clontech, Saint-Germain-en-Laye, France) was fused to a FRT-flanked neomycin cassette (pL451, NCI at Frederick, ncifrederick.cancer.gov). Additional, two sequences of 55 bp each homologous to the target site at the ATG initiation codon of exon 1 of the GAD65 gene were fused to the 5’ and 3’ site of the tdTomato-FRT-neo-FRT fragment. This targeting construct was inserted in the BAC containing the GAD65 gene using homologous recombination (pRed/ET system, GeneBridges, Heidelberg, Germany; Fig 1). Finally, the selection cassette for neomycin resistance was removed by Flp mediated recombination (pFlp, GeneBridges; Fig 1).

BACs were verified using PCR and sequence analysis. PCR reaction was performed with the primers Ex1s and Ex1as (described above) that were placed within the BAC flanking the modification site. PCR products were sequenced to verify sequence identity. Furthermore, the modified BAC was checked for integrity using PCR reaction of selected areas in the 5’- and 3’-regions. The successfully modified BAC was linearized using PI-SceI and purified using gel filtration. Purified linearized DNA was checked by pulse field gel electrophoresis and used for pronuclear injection in fertilized C57BL/6J oocytes. From these injections, 31 mice were born, of which 11 had integrated the transgene and showed germline transmission. Out of these 11 lines, preliminary analysis indicated that two lines showed a rather strong expression, only one of which showed the expected pattern of tdTomato expression. This line was analysed in detail in this study. Mice will be available to the scientific community on request (contact JH).

### Genotyping

Genomic DNA from tail biopsies was isolated with the Invisorb Spin Tissue Mini Kit according to the manufacturer’s instructions (Stratec Biomedical, Birkenfeld, Germany). For PCR analysis, primers were used complementary to BAC Exon 1 sequence 5’ of the translation start signal (sense primer, GTGCAGGGTCGAGGCAAAGGCA) or to the tdTomato sequence (antisense primer, ggacaggatgtcccaggcgaag) yielding a product of 259 bp. PCR was performed with the GoTaq DNA Polymerase (Promega, Mannheim, Germany) according to the manufacturer’s instructions.

### Real-Time PCR Analysis

To establish the number of transgene copies integrated in the genome of the newly generated transgenic mouse line, quantitative Real-Time PCR was applied allowing to compare the number of GAD65 gene containing DNA copies in the transgenic line to the known number of two copies in wild type mice. Therefore, primers were located close to but not within the modified region of the GAD65 gene within the promoter region of the endogenous gene. Short FAM-labelled hydrolysis probes (UPL) containing locked nucleic acid were used for qRT-PCR reactions (Roche Diagnostics GmbH, Mannheim, Germany). Primers and the UPL-probe were designed by ProbeFinder version 2.50 for Mouse (GAD65: sense: cgaggatcacggctactcc; antisense: tgcgtgtttgtatgttccagt; UPL 99; amplicon 91 bp). The genomic sequence of Nrg1 was used to calibrate for the amount of DNA (Nrg1: sense: ggctataatgctaacacagtccaa; antisense: agtggatcgtaacaacactgtca; UPL 38; amplicon 61 bp). Mouse genomic DNA was isolated from tail biopsies of six C57BL/6J mice and six TgN(GAD65-tdTomato)-mice with Invisorb Spin Tissue Mini Kit. 10 ng to 25 ng of genomic DNA was applied per PCR reaction run on a Light Cycler 480 system (Roche Diagnostics GmbH, Mannheim, Germany) according to the manufacturer’s instructions with a pre-incubation step at 95°C for 10 minutes, followed by 45 cycles at 95°C for 10 seconds, 60°C for 10 seconds, and 72°C for 5 seconds. Each sample was run in three technical replicates. The relative amount of GAD65-DNA was calculated by the ddCt method (Software Version LCS480 1.5.0.39, Roche Diagnostics GmbH, Mannheim, Germany).

### Immunohistochemistry

Adult mice were transcardially perfused with 4% paraformaldehyde in phosphate buffered saline (PBS: 137 mM NaCl, 2.7 mM KCl, 8 mM Na_2_HPO_4_, 0.15 mM KH_2_PO_4_, pH 7.4) under deep anesthesia with diethylether. The brain was removed and post-fixed for 24 h in the same fixative. Younger animals were decapitated, the brains removed from the skull and immediately fixed in 4% PFA / PBS for 24 h. 40 μm thick sections were cut on a vibratome. Free-floating sagittal sections of the whole brain were incubated overnight at 4°C with the primary antibodies [mouse anti-GAD65, 1:1000, Millipore (Cat.-No. mab351); rabbit anti-Parvalbumin, 1:5000, Swant (Cat.-No. PV25); rabbit anti-Calretinin, 1:1000, Millipore (Cat.-No. AB5054); rabbit anti-Somatostatin-14, 1:250, Peninsula Laboratories, LLC (Cat. No. T-4103)] diluted in blocking reagent (2% normal goat serum in PBS or 2% normal goat serum / 0.2% Triton X-100 in PBS). Sections were washed three times for 5 min in PBS and incubated for 2 h at room temperature with the secondary antibodies (goat anti-rabbit Cy5 or goat anti-mouse Cy5; 1:1000, Dianova/Jackson Immuno Research, Cat.-No. 111-175-144 or 115-175-166, respectively). After washing with PBS, sections were incubated in PBS containing 1 μg/ml DAPI (Roth, Cat.-No. 6335.1) for 5 min to stain cell nuclei, washed with PBS and finally mounted in Immu-Mount (Thermo Scientific). Confocal images were acquired using a Zeiss LSM700 Axio Observer laser scanning microscope. For quantification, all experiments were performed on slices of three animals (n = 3). Counting of cells was performed using ImageJ on three sections per animal, of which three to four areas (640 μm x 640 μm) per section and brain region were imaged. Epifluoresence images of the whole brain and selected brain regions were recorded using an Axio Observer.Z1 microscope equipped with a motorized stage (Zeiss, Oberkochen, Germany). For detection of EGFP- and tdTomato-fluorescence with 2-photon excitation microscopy a TriMScope microscope (LaVision BioTec, Bielefeld, Germany) with non-descanned detection by GaAsP photomultipliers (Hamamatsu, Hamamatsu City, Japan) was used as described earlier [35]. Two-photon excitation was achieved with a Ti:Sapphire Laser (MaiTai BB, SpectraPhysics, Santa Clara, CA, USA) at 860 nm for fluorescence signals of GlyT2-EGFP expressing glycinergic neurons that were detected through a 531/40 nm band pass emission filter, whereas tdTomato-fluorescence of GABAergic neurons was detected through a BP 641/75 nm band pass emission filter (AHF Analysentechnik AG, Tübingen, Germany) with excitation of 720 nm. Z-stacks (10 μm in total, 1 μm steps) were scanned using a piezo-focus (Physik Instrumente, Karlsruhe, Germany). All settings were controlled by “Imspector” software (LaVision BioTec, Bielefeld, Germany).

### Data processing and presentation

Microscopic images were processed using Zeiss Axiovision software, Zeiss ZEN software, ImageJ and Adobe Photoshop CS2. Pie charts and bar graphs were generated using Microsoft Excel. All quantitative data in the text and shown in the figures are given as mean ± standard error of the mean. Final illustrations were arranged using Corel Draw X4 Graphic.

## Results

### Expression pattern of tdTomato in TgN(GAD65-tdTomato) mice

To label GABAergic interneurons in the mouse brain by a red fluorescent protein, a mouse line expressing tdTomato under the control of the GAD65-gene was generated using transgenesis based on bacterial artificial chromosomes (BAC). The tdTomato open reading frame as well as an additional poly-adenylation site were inserted into the BAC clone RPCI 23-407K8 (Fig 1A–1C), which contains all exons of the GAD65 gene flanked by 72 kb and 64 kb in the 5’- and 3’- direction, respectively. To obtain a transgene as similar as possible to the endogenous gene, the start codon of tdTomato was inserted at the exact position of the start codon of the endogenous Gad65 gene within exon 1. Successful modification as well as identity and integrity of the BAC-construct was verified by PCR (Fig 1D) and sequencing (data not shown).

After microinjection of the BAC-GAD65-tdTomato construct into mouse oocytes, 11 transgenic founder mice were born. Out of these 11 lines, seven lines showed tdTomato fluorescence only in the olfactory bulb in variable extends (data not shown) and only one mouse line showed bright expression of tdTomato in all expected regions of the brain. This line was further analyzed in detail. Using quantitative PCR the number of BAC copies integrated into the genome of this mouse line was determined. Compared to wild type mice, heterozygous transgenic mice contained 2.6 ± 0.02 fold the amount of DNA of the GAD65 promoter region (n = 6 mice). As wild type mice contain two copies of the gene, this result reveals that heterozygous transgenic mice harbor 3 copies of the modified BAC.

**Fig 2:**
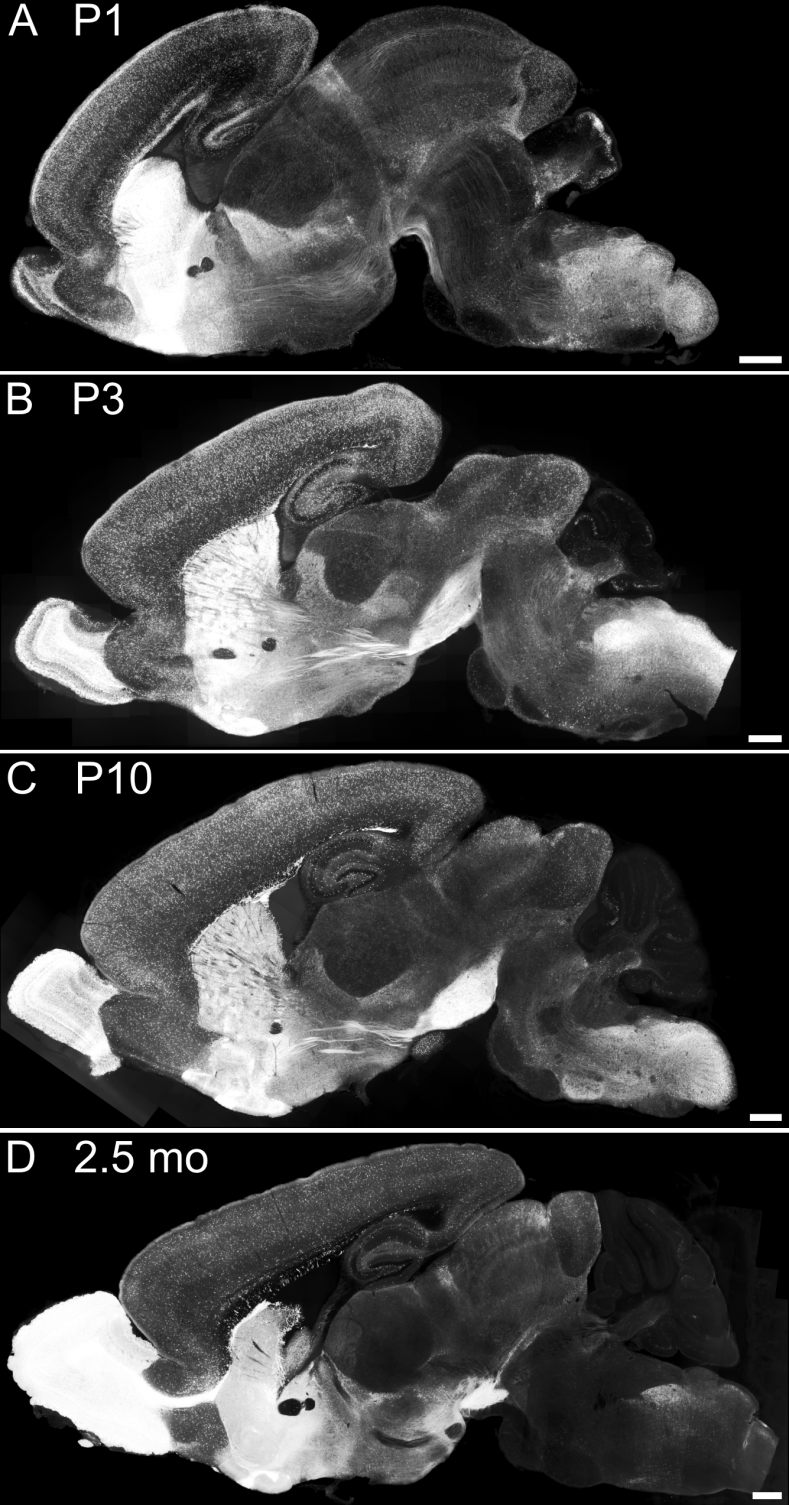
Overall expression pattern of tdTomato in GAD65-tdTomato transgenic mice at different developmental stages. In sagittal brain slices, a high reporter expression was observed in the olfactory bulb and striatum as well as in cortex, hippocampus, brainstem and substantia nigra in all investigated developmental stages (A: P1, B: P3, C: P10, D: adult 2.5 months). Fluorescence intensity of cells varied highly within one brain region and between different areas of the brain as well as different developmental stages. Scale bars: 500 μm.

**Fig 3:**
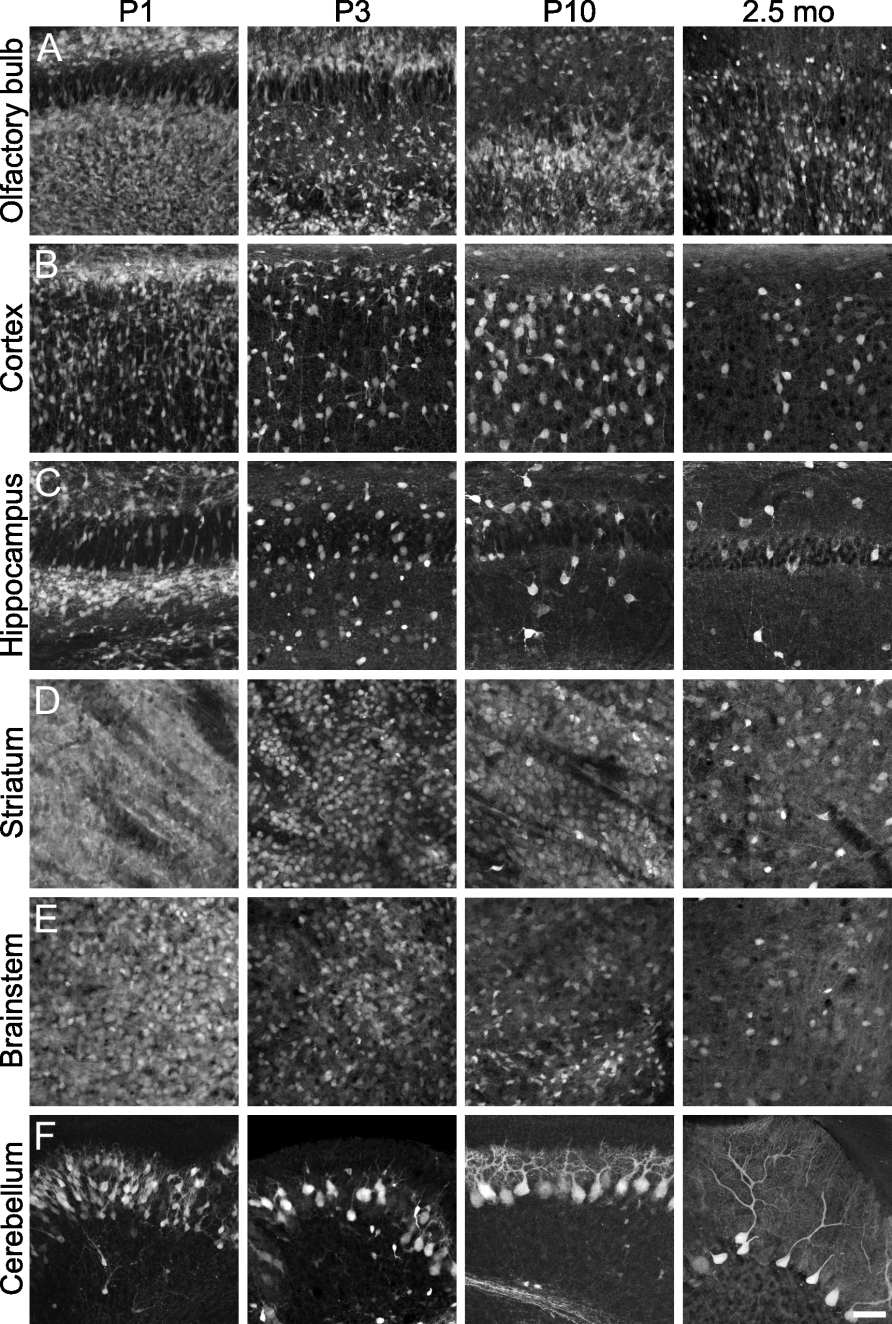
TdTomato expression in various brain regions of GAD65-tdTomato mice at different developmental stages (P1; P3; P10; 2.5 months). **A:** olfactory bulb; **B:** cortex; **C:** hippocampus; **D:** striatum; **E:** brainstem; **F:** cerebellum. TdTomato is expressed in numerous GABAergic neurons within these brain regions and is visible in somata, dendrites, as well as axons. The scale bar corresponds to 50 μm and applies to all panels.

Expression of tdTomato was investigated at different developmental stages (P1, P3, P10 and 2.5 months; Fig 2). As expected, the strongest fluorescent signals were observed in the olfactory bulb and the striatum, i.e. regions with a high number of GABAergic neurons, in all developmental stages (Figs 2 and 3A, 3D). Numerous tdTomato-positive neurons were detected in the cortex within all cortical layers (Figs 2 and 3B) as well as in the hippocampus (Figs 2 and 3C). The fluorescent protein was also expressed within the area of the superior colliculus, inferior colliculus and midbrain. Many cells in the brainstem showed bright fluorescence in younger transgenic mice, whereas in adult animals, a dense net of neurites was observed in addition to neuronal somata (Fig 3E). TdTomato fluorescence was visible in dendrites, somata and axons of neurons, indicating that the protein is distributed throughout the cells. Of note, the intensity of tdTomato fluorescence reflecting the amount of tdTomato expression showed a wide variety from rather dark to very bright cells in all brain regions (Fig 3), consistent with the known variability in expression levels of the endogenous GAD65 gene [16,18].

**Fig 4:**
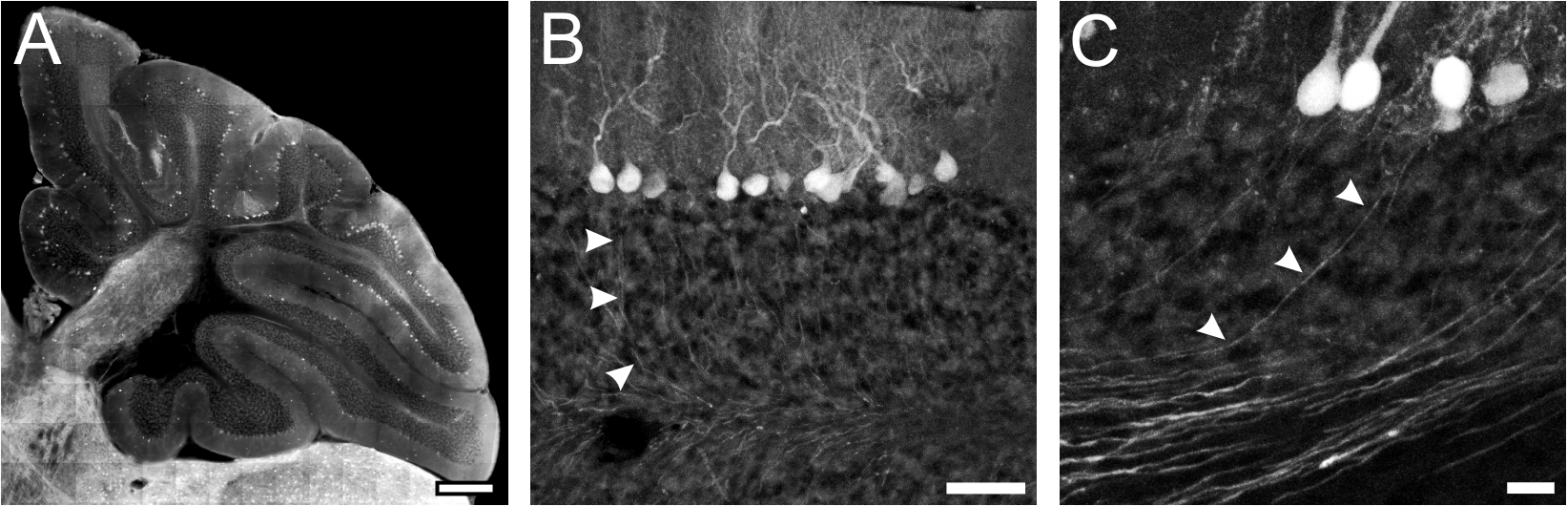
Expression of tdTomato in Purkinje cells in the cerebellum. **A:** Overview of the cerebellum of a 2.5 month old animal showing that only a subpopulation of Purkinje cells expressed tdTomato. **B, C:** In these Purkinje neurons both the dendritic tree as well as the axons (arrowheads) are clearly visible. Scale bars: 500 μm (A), 50 μm (B), 20 μm (C).

A distinct, but less intense expression of tdTomato was observed in the cerebellum. Many immature Purkinje cells as well as few neurons in the molecular layer expressed the fluorescent protein in P1 and P3 mice (Fig 3F). Purkinje cells with a more developed dendritic tree were observed in TgN(GAD65-tdTomato) mice at 10 days of age, and mature Purkinje cells with large dendritic arbors expressed tdTomato in adult transgenic mice (Fig 3F). Remarkably, only a subpopulation of Purkinje cells showed tdTomato expression. Again, their fluorescence intensity differed from cell to cell, but was over all very low in P10 and adult mice compared to cells in other brain regions. Nevertheless, dendritic trees could be clearly visualized, while axons could even be tracked throughout the cerebellum (Fig 4).

**Fig 5:**
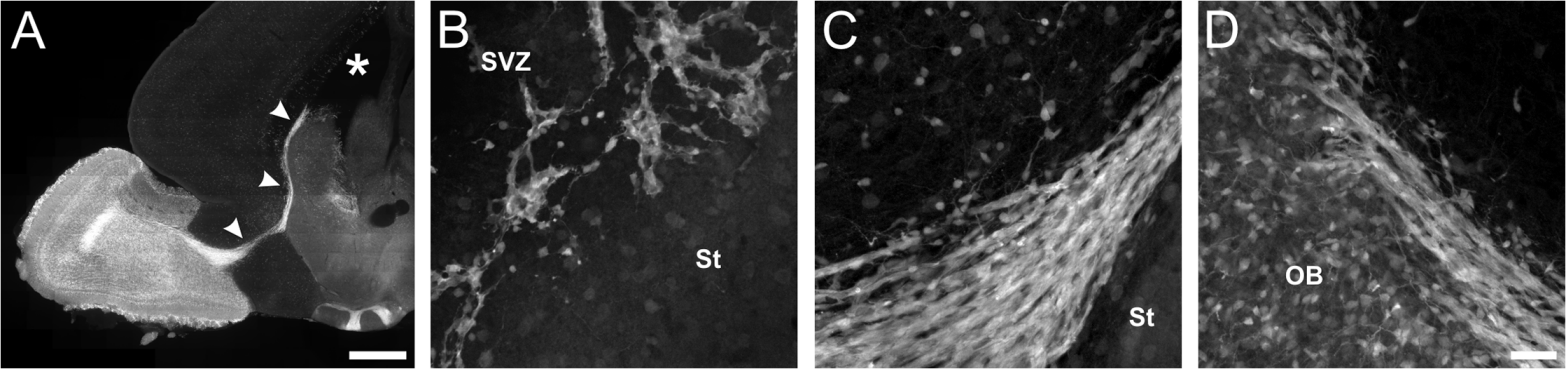
Cells of the rostral migratory stream (RMS) show bright red fluorescence in 2.5 month old GAD65-tdTomato mice. **A:** Overview showing the RMS (arrowheads) from the ventricle (asterisk) to the olfactory bulb. Scale bar: 100 μm. **B-D:** Detailed images of the cells along the RMS migrating from the subventricular zone (SVZ, B) via the rostral forebrain (C) to the olfactory bulb (OB, D). Scale bar in D corresponds to 40 μm and applies to B-D. St: striatum.

**Fig 6:**
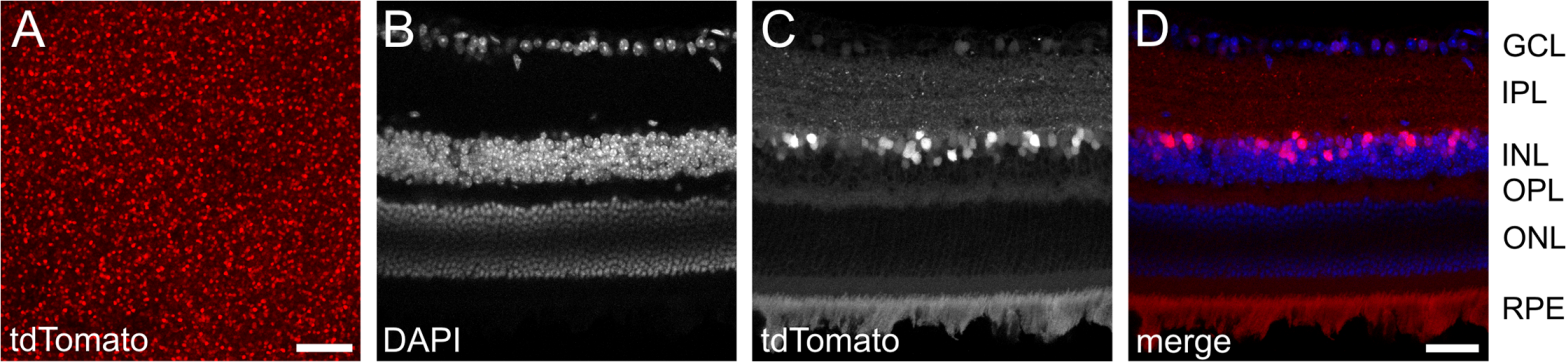
Expression pattern of tdTomato in the mouse retina. **A:** Top view of a retinal wholemount preparation showing the distribution of tdTomato-expressing cells within the retina of a 2.5 month old animal. Scale bar: 100 μm. **B-D:** Transverse section of the retina stained with DAPI to stain cellular nuclei to visualize retinal layering (B). TdTomato-expressing cells (C) were found in inner nuclear layer (INL) and ganglion cell layer (GCL). **D:** overlay of DAPI- (blue) and tdTomato- (red) fluorescence. Scale bar in D corresponds to 40 μm and applies to B-D. IPL: inner plexiform layer; OPL: outer plexiform layer; ONL: outer nuclear layer; RPE: retinal pigment epithelium. Red fluorescence within the RPE is due to autofluorescence.

Neural progenitor cells born within the subventricular zone migrate through the well-defined rostral migratory stream (RMS) from the subventricular zone into the olfactory bulb where they differentiate into GABAergic interneurons, granule cells and periglomerular cells, throughout life [36–38]. Bright red fluorescent cells were observed along this pathway in adult transgenic mice (Fig 5), consistent with the notion that these cells already express GAD65 during migration [39]. Finally, horizontal cells as well as a subpopulation of amacrine cells in the retina are GABAergic interneurons [40,41]. Also expression of GAD65 has been demonstrated in a subset of these cells [41–43]. Consistent with this knowledge, neurons expressing tdTomato were observed in the inner nuclear layer of the retina of TgN(GAD65-tdTomato)-mice (Fig 6). As most of the cell bodies of these cells were located within this layer on the side towards the ganglion cell layer, these cells are most likely amacrine cells. Additional, few tdTomato-expressing cells that showed very low fluorescence intensity were observed in the ganglion cell layer (Fig 6) in agreement with previous studies identifying a small subset of ganglion cells as GABAergic neurons [44,45].

### Almost all tdTomato-expressing cells express endogenous GAD65-protein

**Fig 7:**
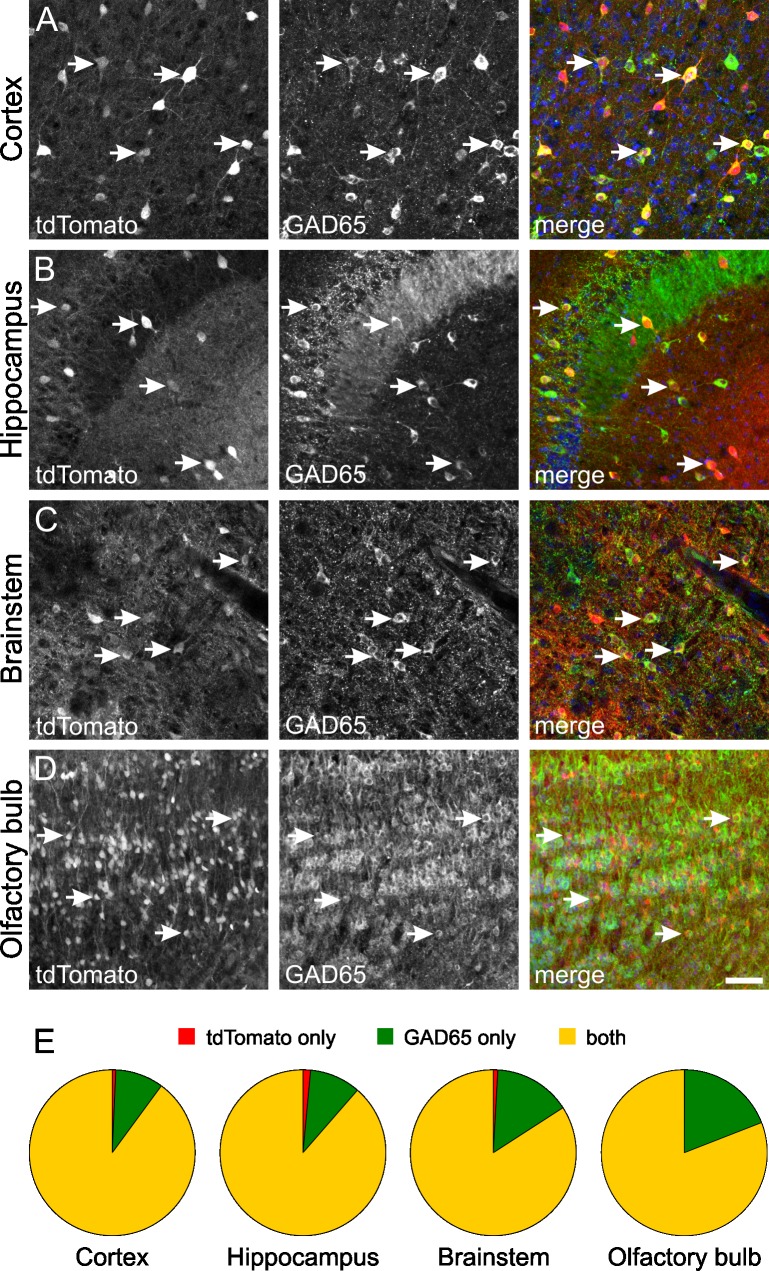
Immunohistochemical verification of tdTomato-expressing cells as GAD65-positive interneurons in GAD65-tdTomato mice. An antibody raised against GAD65 (green) was used to identify tdTomato-positive cells as GABAergic neurons in 2.5 month old mice. **A:** cortex; **B:** hippocampus; **C:** brainstem; **D:** olfactory bulb. All nuclei were stained with DAPI (blue). Arrows highlight examples of cells expressing both tdTomato and GAD65. The scale bar corresponds to 50 μm and applies to all panels. **E:** Quantification of cells expressing tdTomato in the absence of GAD65-immunoreactivity (red); GAD65-positive cells lacking tdTomato fluorescence (green) as well as neurons simultaneously expressing tdTomato and endogenous GAD65 (yellow). While 10% to 20% of GAD65 expressing neurons do not express tdTomato, almost all cells showing tdTomato fluorescence are indeed GAD65-positive GABAergic neurons.

To verify that tdTomato-expressing neurons are indeed GABAergic, GAD65-positive neurons, coexpression of tdTomato and endogenous GAD65-protein was investigated using immunohistochemistry (Fig 7A–7D) and quantified for cortex (Ctx), hippocampus (HC), brainstem (BS) and olfactory bulb (OB) of adult transgenic mice by counting cells expressing only tdTomato, only endogenous GAD65-protein as well as neurons double-positive for tdTomato and GAD65 (Fig 7E). Within these brain regions, most neurons were double-positive for tdTomato fluorescence and GAD65 immunostaining (Ctx: 89.9 ± 3.7%; HC: 88.6 ± 1.8%; BS: 84.2% ± 2.2%; OB: 81 ± 0.7%; all mean ± SEM; n = 3 mice; total number of cells counted: Ctx: 4788; HC: 1593; BS: 949; OB: 2021). Only a small percentage of cells showed GAD65 immunoreactivity without tdTomato fluorescence (Ctx: 9.4 ± 3.6%; HC: 10.0 ± 2.2%; BS: 15.0% ± 1.7%; OB: 18.9 ± 0.8%). Most importantly, only a very small number of cells expressed tdTomato in the absence of detectable levels of endogenous GAD65-protein (Ctx: 0.7 ± 0.2%; HC: 1.4 ± 0.8%; BS: 0.8% ± 0.6%; OB: 0.1 ± 0.2%). These data imply that—while not all GABAergic cells are labelled by tdTomato expression—cells expressing tdTomato are indeed GABAergic neurons (Fig 7E).

### TdTomato-positive cells express parvalbumin, calretinin and somatostatin

**Fig 8:**
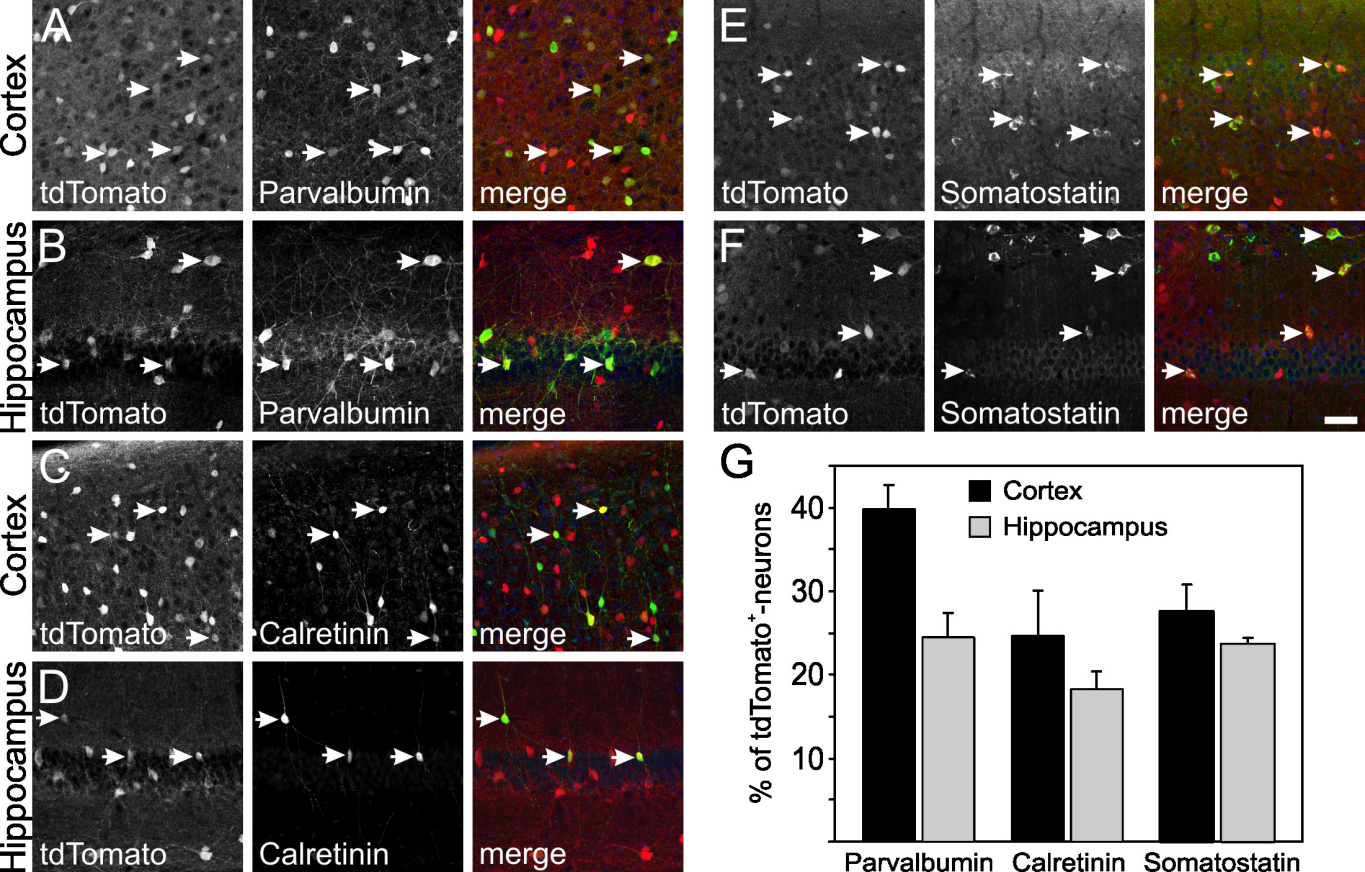
Identification of parvalbumin-, calretinin- and somatostatin-expressing subpopulations of interneurons among tdTomato-expressing neurons in transgenic mice. Immunohistochemical analysis showed the colocalization of the interneuron markers parvalbumin (A, B), calretinin (C, D) or somatostatin (E, F) with tdTomato fluorescence (red) in the cortex (A, C, E) and hippocampus (B, D, F) of 2.5 month old mice. Right panels show the overlay of tdTomato (red), parvalbumin, calretinin or somatostatin (green) as well as nuclei stained with DAPI (blue). Arrows highlight examples of cells expressing both tdTomato and either parvalbumin, calretinin or somatostatin. The scale bar corresponds to 50 μm and applies to all panels. **G:** Quantification of the relative contribution of parvalbumin-, calretinin- and somatostatin-expressing neurons to the number of cells expressing tdTomato.

GABAergic neurons are a highly diverse group of neurons, which can be further classified by many different parameters [1,46]. One parameter discriminating subpopulations of GABAergic neurons is the expression of proteins like parvalbumin, calretinin and somatostatin [47,48]. Therefore, to further characterize the tdTomato-expressing cells in adult TgN(GAD65-tdTomato)-mice, immunohistochemical analysis with antibodies for parvalbumin, calretinin and somatostatin was performed and quantified (Fig 8). In the cortex of TgN(GAD65-tdTomato)-mice, 39.8 ± 2.8% of tdTomato-expressing cells coexpressed parvalbumin, 24.6 ± 5.4% calretinin, and 27.6 ± 3.2% somatostatin (n = 3 mice; Fig 8G). In the hippocampus, the fraction of double-positive cells was lower than in the cortex. 24.5 ± 2.7% of tdTomato-expressing neurons coexpressed parvalbumin, 18.3 ± 2.0% were double-positive for tdTomato and calretinin, and 23.7 ± 0.8% were somatostatin-positive neurons.

### TdTomato-positive cells can be discriminated from EGFP expressing cells using different microscopic techniques

**Fig 9:**
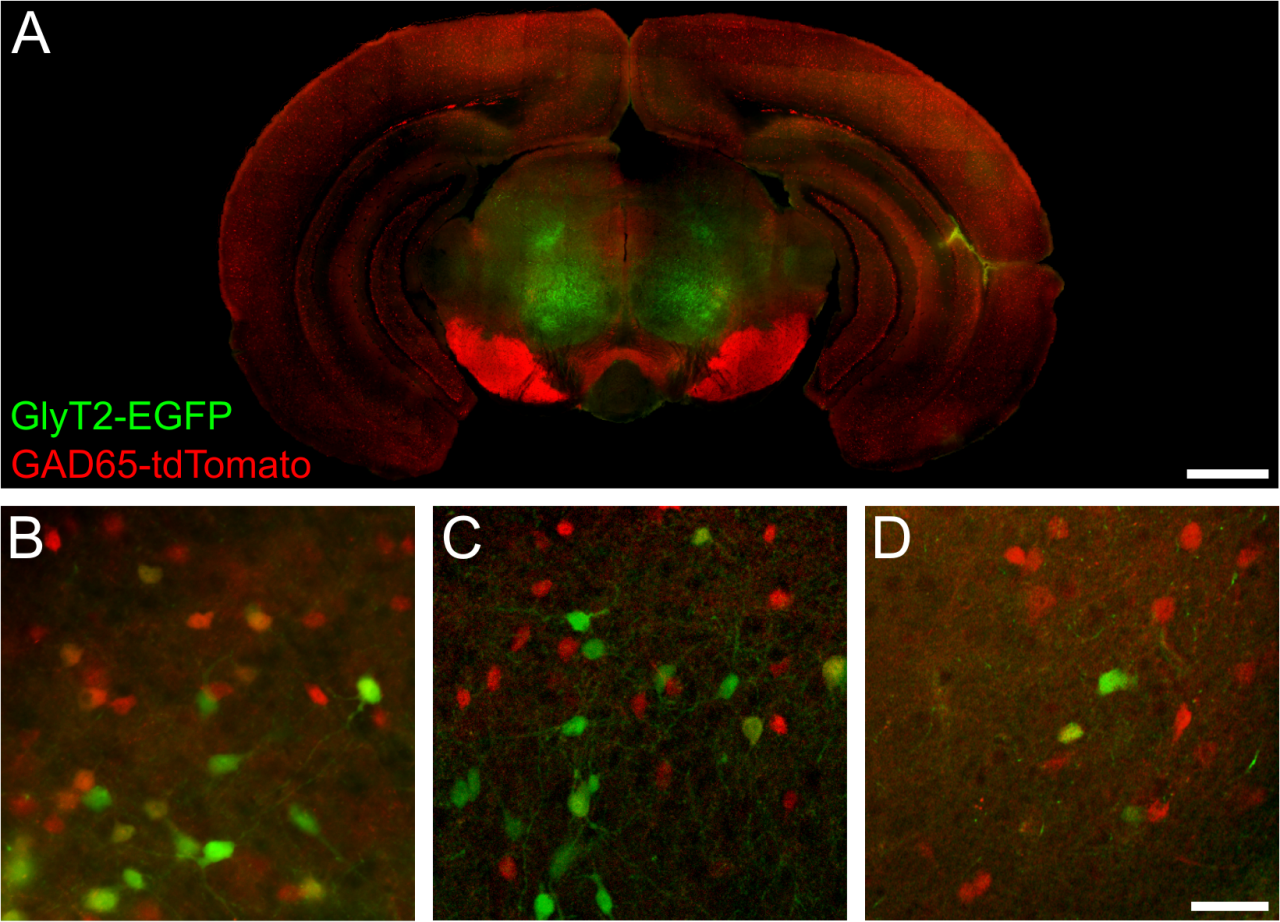
Identification of GABAergic and glycinergic neurons in TgN(GAD65-tdTomato) x TgN(GlyT2-EGFP) double transgenic mice by several microscopic techniques. **A:** Overview of a frontal brain slice of a double transgenic 30 day old mouse showing tdTomato (red) expressing GABAergic and EGFP (green) expressing glycinergic neurons. The image was acquired using epifluorescence. Scale bar: 1 mm. **B:** High magnification epifluorescence image. **C:** GABAergic and glycinergic cells expressing tdTomato or EGFP, respectively, can also be visualized using confocal imaging. **D:** Also by using 2-photon laser scanning microscopy, tdTomato- and EGFP-fluorescence can be observed allowing for unequivocal identification of GABAergic and glycinergic neurons, respectively. Scale bar in D corresponds to 40 μm and applies to B-D.

The rational for generating the TgN(GAD65-tdTomato)-mice was to allow for co-labelling of a different population of cells expressing for example EGFP. As proof of principle, we crossbred TgN(GAD65-tdTomato)-mice to TgN(GlyT2-EGFP) animals [25], which express the green fluorescent protein EGFP in glycinergic neurons. GABAergic and glycinergic neurons expressing tdTomato or EGFP, respectively, can clearly be identified at low and high magnification (Fig 9). Some cells showed expression of both fluorescent proteins (Fig 9), consistent with the notion that some inhibitory neurons throughout the brain simultaneously use GABA and glycine as transmitter [7,49–52]. Furthermore, the different cell populations can be distinguished using several microscopic techniques including epifluorescence microscopy, confocal- as well as 2-photon laser scanning microscopy (Fig 9B–9D), thereby highlighting the manifold potential applications of the newly generated TgN(GAD65-tdTomato)-mouse line.

## Discussion

BAC-transgenic mice are an excellent tool to study different cell populations within complex tissues as they allow the expression of reporter proteins with expression patterns similar to the endogenous genes. The newly generated BAC-transgenic mouse line TgN(GAD65-tdTomato) described here showed a stable and bright expression of the red fluorescent protein tdTomato exclusively in GABAergic neurons in all expected regions of the brain as well as in different developmental stages. TdTomato fluorescence was observed e.g. in the olfactory bulb, striatum, cortex, hippocampus, superior and inferior colliculus, brainstem as well as in projections from the striatum to the substantia nigra. The observed distribution of tdTomato-expressing cells is consistent with previous results from studies investigating the distribution of GAD65 and GAD67 mRNA using *in situ* hybridization [11,16,18,19].

The identity of tdTomato-expressing cells was verified using immunohistochemical staining for the endogenous GAD65 protein as a reliable marker for GABAergic neurons. Most importantly, only very few cells showed tdTomato fluorescence in the absence of detectable GAD65 immunoreactivity (0.1% – 1.4% in the brain regions investigated; Fig 7E), indicating that expression of tdTomato within a cell allows identification as a GABAergic cell with a very low error rate. Is tdTomato expressed in all GABAergic cells? To address this question, tdTomato-expressing cells were quantified in relation to all cells expressing endogenous GAD65-protein. 80% to 90% of GAD65-positive cells expressed tdTomato, indicating that indeed a high proportion of GABAergic cells are labelled by tdTomato fluorescence. However, this proportion might be slightly overestimated as some GABAergic neurons might only express GAD67 in the absence of GAD65, thereby lacking also tdTomato expression. However, in situ hybridization studies reported for only three brain regions (globus pallidus, ventral pallidum, lateral mammillary nucleus) a higher density of GAD67-positive cells compared to GAD65-positive cells, indicating that almost all GABAergic neurons express mRNA for both GAD65 and GAD67 [16]. Therefore, it is likely that the percentage of tdTomato-positive cells in relation to all GABAergic cells is only slightly—if at all- overestimated. TdTomato fluorescence intensity varied strongly between different brain regions indicating different activity of the transgenic GAD65-promoter, which most likely correlates with expression levels of endogenous GAD65 in these areas. Furthermore, different fluorescence intensities were even observed between neurons within a given brain region, e.g. in cortex, hippocampus and brainstem. It remains to be established whether this heterogeneity in expression of tdTomato in the newly generated transgenic line relates to different subtypes of GABAergic interneurons and/or to different physiological properties or different expression patterns also of other genes [46,48,53–55]. However, TgN(GAD65-tdTomato) mice will be a valuable tool to identify and investigate these subpopulations as well as the underlying regulatory mechanisms in various regions of the mouse brain.

GABAergic neurons are not a homogenous population of cells and have been classified in subpopulations using several parameters including electrophysiological properties, release of neuropeptides or expression of certain genes like calcium binding proteins [46,48,53–55]. To further characterize the cells expressing tdTomato in the TgN(GAD65-tdTomato)-mouse line, immunohistochemistry was applied to identify parvalbumin-, calretinin- and somatostatin-positive cells among tdTomato-expressing neurons in the cortex and hippocampus of adult mice. Parvalbumin was expressed in 40% of tdTomato-positive cortical neurons, while 25% coexpressed calretinin and about 28% somatostatin (Fig 8G). These results are well in line with results from mouse cortex showing that 39% of GABAergic cortical interneurons express parvalbumin while 16% to 24% are calretinin-positive and 23% coexpress somatostatin [47,56]. In the hippocampus, 25% of tdTomato-expressing neurons coexpressed parvalbumin, 18% calretinin and 24% were double-positive for tdTomato and somatostatin (Fig 8G) consistent with data obtained on rat hippocampus reporting 23% parvalbumin-positive as well as 28% calretinin-positive and 23% to 45% somatostatin-positive cells among GABAergic neurons [57,58]. These results indicate that while most, but not all GABAergic neurons express tdTomato (see above), a representative selection of cells is labelled by tdTomato, at least in respect to the coexpression of these marker proteins.

Besides this general assessment of the TgN(GAD65-tdTomato) mouse line, a few special features deserve further attention:

1. In the cerebellum, transgenic expression of tdTomato was observed only in a subset of Purkinje cells despite the well-established expression of mRNA of both GAD isoforms in these cells [18,19]. In some cerebellar lobes many Purkinje cells expressed tdTomato whereas in other lobes expression of tdTomato was absent (Fig 4A). While the reason for this heterogeneous expression is unclear, similar patterns have been described for both expression of endogenous genes like Aldolase C [59] and “early onset markers” like En-2 or Wnt-7b [60,61] as well as for transgenic expression using e.g. the Thy1-promoter [34]. However, the rather sparse expression in Purkinje cells is a strong advantage for imaging structures like axons and dendrites of these cells, as expression in all cells would prohibit identification and tracking of processes of a certain cell.
2. Proliferation of neural precursor cells in the SVZ of the lateral ventricles persists throughout life in different species and neurons born in the SVZ migrate through the RMS to the olfactory bulb [62–65]. These cells express both GAD65 and GAD67 [39,66]. Consistently, bright tdTomato fluorescence was detected in cells along the rostral migratory stream in brains of TgN(GAD65-tdTomato)-mice at different developmental stages, suggesting that these mice might be also an advantageous tool for research on this neurogenic niche as well as the fate and properties of those cells.
3. Finally, tdTomato fluorescence was observed within the retina, where weak fluorescence was detected in ganglion cells, while much brighter fluorescence was found within cells in the inner nuclear layer, being most likely amacrine cells. Expression of endogenous GAD65 has indeed been reported for ganglion cells and amacrine cells in rats, cats, zebrafish and guinea pigs [42,44,45,67–69], indicating that also in the retina the expression of tdTomato reliably reflects the expression pattern of endogenous GAD65.

Taken together, the newly generated TgN(GAD65-tdTomato)-mouse line allows unequivocal identification of around 80% to 90% of all GABAergic cells by bright fluorescence of tdTomato. The expression of a fluorescent protein in the red range of the spectrum is unique among existing transgenic mouse lines labelling GABAergic neurons. Therefore, this mouse line will allow for the first time concurrent labelling of GABAergic neurons and a second cell population highlighted by expression of the green fluorescent protein, thereby greatly facilitating studies e.g. of dynamic morphological interaction of other cells with GABAergic neurons.
